# Influence of Climate on Pectoral Diseases

**Published:** 1876-03

**Authors:** J. G. Westmoreland

**Affiliations:** Atlanta, Ga.


### INFLUENCE OF CLIMATE ON PECTORAL DISEASES.

By J. G. WESTMORELAND, M.D., Atlanta, Ga.

In determining a proper course of general management for invalids, it is proper first to inquire what kind of constitutional influences are required. In diseases whose cure is not looked for in the use of pharmacological remedies alone, but also in climatic and other external influences, physicians are expected to advise, as well as in those affections under the control of medicines. The medical adviser is presumed to know whether, in a particular case, relaxing, sedative, and soothing influences, or innervation and general invigorating means are demanded.

Chronic affections of the liver, bowels, spleen, brain, and perhaps the kidneys and bladder, are more common in warm latitudes; while diseases of the respiratory organs are said to abound in cold climates. Constitutional conformation or temperament doubtless predisposes to the existence of certain diseases. In a strumous constitution, for instance, scrofula or tuberculosis is developed under favorable circumstances, and disturbance of the liver and bowels are more likely to occur in persons of bilious temperament. These opposite states of constitutional conformation require different management to prevent and to cure the respective diseases to which they are subject. A home in the tropics is better for one, while in the other, biliary and bowel affections may be avoided by a residence in a frigid zone.

Other facts, connected with hygiene and the cure of disease, must be considered. Without taking into the account all the indirect influences favorable and unfavorable to the preservation and restoration of health, the medical adviser fails to discharge his duty. The health of other organs than that diseased or threatened with disease, must be considered. While we protect from direct injurious influences, it must be remembered that we sometimes indirectly hasten the dreaded difficulty by depressing a controlling organ of the body, by the means employed. Thus, when the subject of strumous constitution visits a warm climate to prevent the injurious effects of cold weather upon the respiratory apparatus, he meets with enervation and depression from the warm, relaxing temperature, which state of the nervous system tends materially to the development and unfavorable progress of tuberculosis. It is well known that invigoration of the nervous system is indispensible in the treatment of tuberculous subjects, and in shunning the irritation of colder air, the patient is exposed to general enervation, equally injurious. In steering safely from Scylla he strikes the fatal strand of Carybdis.

Moreover, oily food, so useful in one of strumous diathesis, is rarely relished to the necessary amount, in warm climates, to to afford adequate support. Indeed, consumptives seeking health in Southern latitudes, rely too exclusively on the change of climate for relief, and often the fatal termination is hastened by such a course. Again, many stop short of reaching a location free from chilling winds, and hence are exposed to the enervating influence of a burning sun and the bronchial irritation of "cold snaps," which occasionally occur in many places visited by such invalids. Even chronic bronchitis, in which full nervous vigor is not so important to recovery as in tuberculosis, is protracted by such changes.

In view of these facts, and from the result of cases under my direction, visiting Florida in the neighborhood of Jacksonville, I have for many years advised consumptives to remain at home, unless they go sufficiently far south to avoid sudden changes of temperature, to which most of Florida is subject. Even then, without great care in promoting general vigor by nourishing and oily food, and by every means of promoting nervous power, no advantage is offered.

